# A cross-sectional study on resilience and death anxiety among emergency nurses

**DOI:** 10.1186/s12912-025-02980-7

**Published:** 2025-04-15

**Authors:** Ayman Mohamed El-Ashry, Haitham Mokhtar Mohamed Abdallah, Shimmaa Mohamed Elsayed, Mahmoud Abdelwahab Khedr, Mona Metwally El-Sayed, Mohamed Adel Ghoneam

**Affiliations:** 1 Psychiatric and Mental Health Nursing, Faculty of Nursing, Alexandria University, Alexandria, Egypt; 2 Critical Care and Emergency Nursing, Faculty of Nursing, Alexandria University, Alexandria, Egypt; 3 College of Nursing, Jouf University, Sakaka, Saudi Arabia; 4 Critical Care and Emergency Nursing, Faculty of Nursing, Damanhour University, El Beheira, Egypt; 5 Critical Care and Emergency Nursing, Faculty of Nursing, Faculty of Nursing, Beni-Suef University, Beni-Suef, Egypt

**Keywords:** Resilience, Death anxiety, Emergency nurses, Hierarchical regression analysis

## Abstract

**Background:**

Emergency nurses frequently encounter death and experience significant levels of death anxiety, impacting their mental well-being and professional performance.

**Objective:**

Explore the levels of resilience and death anxiety among emergency nurses and examine the relationship between these two constructs.

**Research design:**

Following STROBE guidelines, a cross-sectional descriptive correlational design was employed, with data collected from 417 emergency nurses in three hospitals.

**Tools:**

The Socio-demographics Data Sheet, the Arabic Scale of Death Anxiety, and the Connor-Davidson Resilience Scale were utilized for data collection.

**Results:**

Results indicated average moderate levels of resilience and death anxiety among participants. A negative correlation was observed between resilience and death anxiety, suggesting that as resilience decreased, death anxiety tended to increase. Significant associations were found between death anxiety and gender, age, and years of experience. The stepwise hierarchical linear regression analysis of substantial factors predicting death anxiety among emergency nurses revealed that resilience, years of experience, and gender were significant predictors of death anxiety, explaining 10.2% of the variance.

**Conclusion and nursing implications:**

These findings underscore the importance of addressing mental health challenges among emergency nurses and highlight the need for interventions aimed at promoting resilience and mitigating death anxiety. By fostering a supportive environment and providing resources for mental health, healthcare institutions can empower emergency nurses to thrive in their demanding profession while delivering optimal care to patients in critical situations.

**Clinical trial number:**

Not applicable.

**Supplementary Information:**

The online version contains supplementary material available at 10.1186/s12912-025-02980-7.

## Introduction

The Emergency Department (ED) is a cornerstone within the healthcare system, operating around the clock to provide urgent and specialized medical attention to individuals confronting acute and life-threatening medical issues. In these settings, healthcare professionals often confront death as they work tirelessly to save lives [1]. Despite their best efforts, not all patients survive, and emergency unit staff must cope with the emotional impact of death, leading to feelings of grief, guilt, and burnout. The fast-paced and high-pressure environment of emergency units can make it challenging for staff to process their emotions surrounding death [2, 3].

Death is a fundamental aspect of life that many tend to avoid, yet it remains a constant presence for healthcare workers, particularly nurses, who regularly confront its realities. While some openly discuss death, others experience death anxiety, unease about mortality shaped by cultural influences [4]. Recent research indicates that nurses, due to their proximity to patients and supportive roles, often experience heightened levels of this anxiety, which can hinder their performance and communication. Stressful situations can exacerbate this preoccupation, leading to persistent thoughts and desires about death, termed death obsession [1, 5]. Factors such as humor in colleagues and patients can influence these feelings, serving as a coping mechanism amid the complexities of mortality [5].

Despite grappling with personal grief, nurses often lack societal acknowledgment or avenues to articulate their sorrow for their patients openly. Furthermore, the fear of mortality impacts both professional and individual dimensions of nurses’ lives, giving rise to adverse sentiments such as anger, frustration, feelings of insignificance, grief, desolation, and even contemplation of self-harm. This circumstance can also influence their approach to work, the caliber of their performance, and their interactions with patients [6]. Individuals with heightened levels of death anxiety often find it challenging to broach the topic of mortality with patients and their families [7, 8].

These professional challenges can impact nurses’ psychological resilience, defined as their ability to recover from life’s challenges. When nurses experience fear or anxiety related to death, they may exhibit less teamwork and struggle to remain composed when communicating with patients about their healthcare [9]. This apprehension towards death may also impact their ability to adhere to professional standards, such as providing spiritual and palliative care to those who are dying [5, 9]. Various factors, including age, gender, and religious beliefs, can influence the extent of death anxiety in nurses. Therefore, maintaining psychological resilience is crucial for nurses to avoid professional and personal burnout [10, 11]. By fostering a supportive environment and providing resources for mental health, healthcare institutions can empower emergency nurses to thrive in their demanding profession while delivering optimal care to patients in critical situations [7, 12].

In emergency care, nurses ensure quality care, particularly for terminally ill patients. Given the emotionally complex nature of this care, nurse should undergo specialized training to cultivate resilience when faced with highly stressful situations. Resilience entails the ability to bounce back from ongoing challenges and self-repair, maintaining psychological stability and health in adversity [5, 13]. In professions such as nursing, prolonged exposure to patients’ trauma and suffering can have significant emotional and psychological impacts. For example, a nurse working in an intensive care unit who frequently witnesses the distress of critically ill patients and their families may develop symptoms similar to post-traumatic stress disorder (PTSD), such as emotional detachment, heightened anxiety, and disrupted sleep patterns [14].

Prolonged exposure to stress has been linked to reduced production of brain neurotrophic factors, heightening susceptibility to symptoms of anxiety and depression. Despite these obstacles, resilience empowers nurses to navigate their work environment and uphold mental well-being. High levels of resilience among healthcare professionals correlate with lower rates of burnout [4, 7, 14]. In nursing, resilience specifically addresses professional challenges, serving as a personal capacity to manage workplace demands. Cultivating resilience is essential for nurses to cope with professional hurdles and safeguard their mental health, transforming negative experiences into positive ones [7, 15].

## Research gap and significance of the study

Emergency care nurses often experience significant physiological and psychological exhaustion, including feelings of professional inadequacy and guilt [16, 17]. These challenges can diminish their psychological resilience, defined as the ability to recover from life’s difficulties and adversities [6]. In the context of death anxiety, resilience refers to the ability to cope with and recover from the emotional and psychological stress associated with frequent exposure to death and dying [13, 18].

This study focuses on the interplay between death anxiety and resilience among emergency nurses. It investigates how resilience can buffer the impact of death anxiety on professional performance and personal well-being. The study also explores the potential for resilience-building interventions to enhance nurses’ ability to cope with the emotional demands of their work, contributing to improved mental health outcomes and patient care quality in emergency settings. By exploring the relationship between resilience and death anxiety levels among emergency nurses, this study fills a gap in the literature. It highlights the importance of further research in this area. It represents a crucial step towards recognizing and addressing the mental health challenges faced by emergency nurses, promoting their long-term well-being and effectiveness in their roles as frontline healthcare providers. This study aimed to examine the relationship between resilience and death anxiety of nurses working in emergency care units.

### Research hypothesis

- Higher level of resilience is associated with lower level of death anxiety among nurses working in emergency care units.
- Female nurses will report higher level of death anxiety compared to male nurses.
- Nurses with fewer years of professional experience will report higher level of death anxiety compared to those with more experience.
- Nurses residing in urban areas will report higher level of death anxiety compared to those in rural areas.

## Study design

This study followed a cross-sectional descriptive correlational design following STROBE guidelines [19]. (Supplementary file 1)

## Setting

The study included three general hospitals serving the Al-Beheira population: Damanhour Medical National Institution Hospital, Itay Elbaroad Hospital, and Kafr Eldawar Hospital. Damanhour Medical National Institution Hospital has an emergency unit with 25 beds and 160 emergency nurses. Itay Elbaroad Hospital has an emergency unit with 15 beds and 150 emergency nurses. Kafr Eldawar Hospital has an emergency unit with 25 beds and 148 emergency nurses. So, the total population included in the study was 458 ICU nurses.

## Study subjects and sample calculation

G*Power Windows 3.1.9.7 program was used to estimate the participants. The parameters were as follows: power (1-β err probability) = 0.99, effect size = 0.25, α-error probability = 0.01, groups’ number = 1, and predictors = 3. The program disclosed 318 nurses to be the sample size [20]. From 458 nurses, a convenience sample selection of 417 emergency nurses was made with a response rate of 91.04%.

**Fig. 1:**
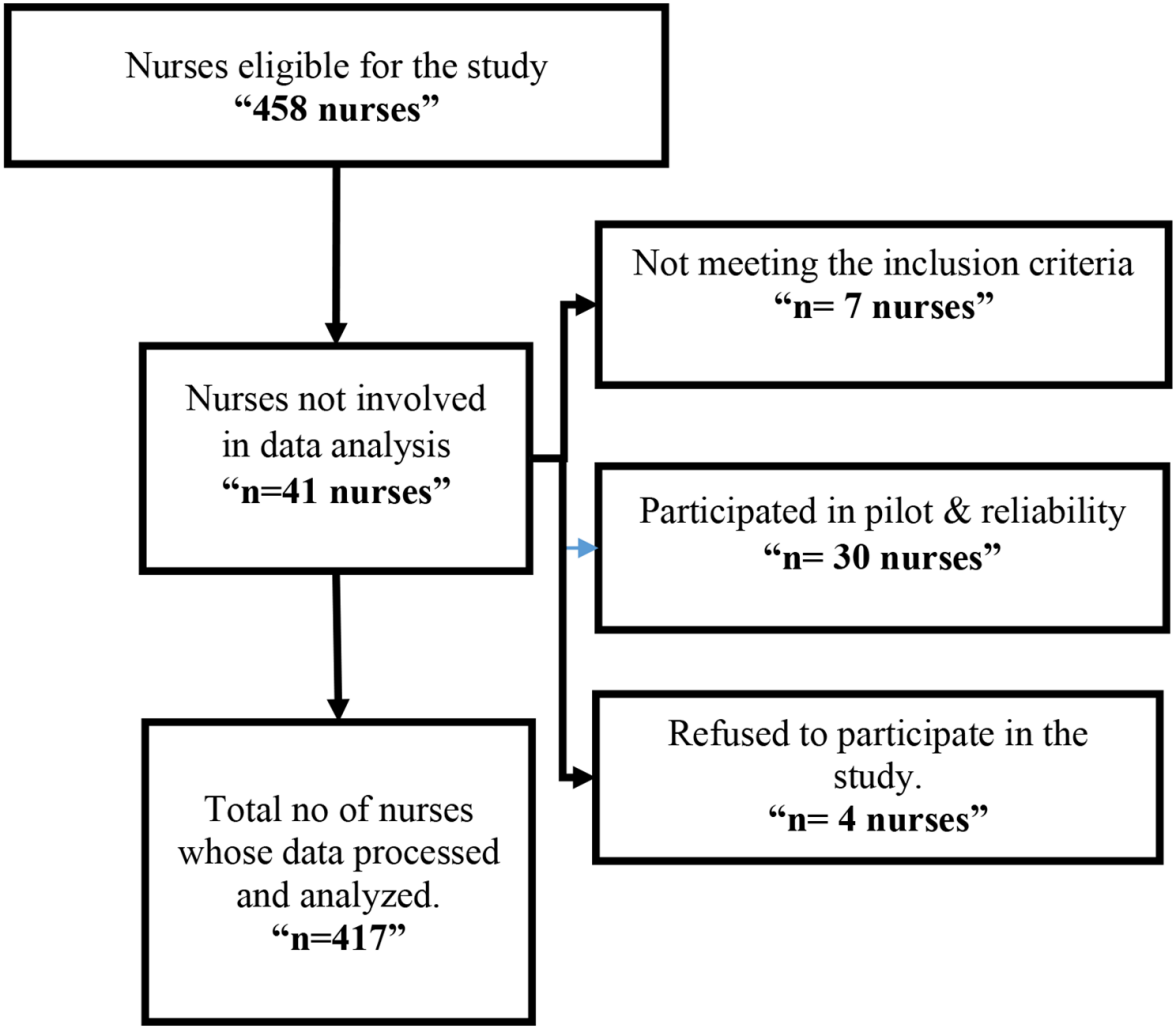
Flow chart of participants’ recruitment process

According to the flow diagram (Fig. 1), 458 nurses met the eligibility requirements to participate in the study. Seven nurses did not fulfill the inclusion criteria, four declined to participate, and thirty had previously participated in the pilot study. Four hundred and seventeen nurses were recruited in the analysis (Fulfilling the required number resulted from G*Power Windows *<* 318 nurses). The inclusion criteria included nurses aged 21–60 who were willing to participate in the study and actively worked during the data collection. Those who were not exposed to patients at data collection time and were not considered valid registered nurses with licenses were excluded from the study.

### Study tools and data collection (supplementary file 2)

#### Tool I: socio-demographics data sheet

considering gender, age, marital status, number of kids, place of residence, level of education, academic standing, department (specialization), years worked after earning a bachelor’s degree in nursing, and years worked in the current role.

#### Tool II: the Arabic scale of death anxiety (ASDA)

ASDA was developed to assess the level of death anxiety in individuals. The scale consists of 20 items, each rated on a Likert scale ranging from 1 (No) to 5 (Very much.). The total score on the scale can range from 20 to 100, with higher scores indicating higher levels of death anxiety. The ASDA was validated in three Arab nations - Egypt, Kuwait, and Syria - and showed strong internal consistency and stability. The alpha reliabilities ranged from 0.88 to 0.93, and the 1-week test-retest reliability (Egyptians only) was found to be 0.90 [21].

#### Tool III: the Connor-Davidson resilience scale (CD-RISC-10)

The CD-RISC is a test that measures resilience, or ability to bounce back after a stressful situation, catastrophe, or trauma [22]. The tool was valid and reliable for the Arabic population, so The Arabic version of the Connor-Davidson Resilience Scale-10 was used in the present study [23]. It comprises 10 items and is graded as follows, with 0 to 4 on a 5-point scale: 0 not true at all, 1 rarely true, 2 sometimes true, 3 often faithful, and 4 accurate nearly all the time. The ratings generate a number between 0 and 40; higher scores suggest greater resilience. The CD-RISC demonstrated test-retest reliability (intraclass *r* = 0.87) and acceptable internal consistency (Cronbach’s alpha = 0.89) [23].

### Ethical approval

The study received legal approval and permission from the Research Ethics Committee of the Faculty of Nursing, Damanhour University (No 93-b) on January 18, 2024. The administrative authorities of the three hospitals and the Ministry of Health in Egypt also approved. The researchers ensured that nurses participating in the study were fully informed about the confidentiality and anonymity of their answers and were aware of the study’s objective. Written consent was obtained after the nurses were informed of their right to decline to participate. Participants were also told that their privacy and confidentiality would be protected, and they could withdraw from the study at any time, even after it had begun.

### Pilot study and reliability

A pilot study assessed the reliability of the questionnaires used in the main study. The pilot study participants were selected through convenience sampling and consisted of 30 emergency nurses [24]. These participants were interviewed individually and face-to-face once, and their responses were excluded from the primary analysis. The reliability of the questionnaires was assessed using Cronbach’s alpha coefficient, a commonly used metric for evaluating internal consistency. The analysis revealed that the Cronbach’s alpha coefficient for the Arabic Scale of Death Anxiety was **0.821**, demonstrating high internal consistency. Similarly, the Arabic version of the Connor-Davidson Resilience Scale yielded a Cronbach’s alpha coefficient of **0.801**, indicating high internal consistency. These results confirm that the questionnaires employed in the main study are reliable tools for measuring the constructs of death anxiety and resilience among emergency nurses.

## Study procedure

### Data collection

After obtaining the necessary permissions, the data collection process began with explaining the research goals to the participating nurses, emphasizing the confidentiality and anonymity of their responses. A representative sample of emergency nurses was recruited through convenience sampling, excluding those who participated in the pilot study. The researcher conducted individual face-to-face interviews with each participant in a quiet area during their breaks or between shifts. Each participant took approximately **15 to 25 min** to complete the questionnaire, ensuring thoughtful and accurate responses without disrupting their work schedules. Data collection occurred over **three months**, from **December 2023 to February 2024**. The questionnaires were distributed and collected face-to-face, allowing the researcher to address questions or concerns immediately. This approach ensured a high response rate and accurate data collection and maintained the privacy and convenience of the participants.

### Data analysis

The data were entered into a computer and analyzed using IBM SPSS software version 23.0. The Shapiro-Wilk test produced a non-significant p-value, confirming that the variables followed a normal distribution. The one-way ANOVA test was utilized for comparisons involving more than two categories. The Student’s t-test was applied to compare two categories for normally distributed quantitative variables. The Pearson correlation coefficient examined relationships between customarily distributed quantitative variables. A hierarchical linear regression analysis was conducted to identify factors influencing death anxiety (ASDA), with Variance Inflation Factor (VIF) values checked to ensure no multicollinearity among predictors. The significance level for all statistical tests was set at *p* < 0.05.

## Results

Table 1 shows the distribution of nurses according to demographic characteristics. Most nurses were female (73.1%) compared to male participants (26.9%). Regarding age distribution, the majority fell within the 20 to less than 30 age bracket (86.1%), with a mean age of 26.94 years and a standard deviation of 4.96 years. Marital status varied among the participants, with the majority being single (71.7%), followed by married individuals (25.2%). In terms of residence, more nurses resided in urban areas (60.0%) compared to rural areas (40.0%). Living arrangements showed that the majority lived with family (89.0%), while a smaller percentage lived alone (4.8%) or with relatives (1.2%) or with others (5%). Regarding professional characteristics, the distribution of nurses based on the number of cardiopulmonary resuscitations (CPR) performed showed that 18.7% had performed less than 50 CPRs, 55.2% had performed between 50 and 100 CPRs, and 26.1% had performed more than 100 CPRs. In terms of years of experience, most of the participants had less than 5 years of experience (72.4%), with smaller proportions having 5 to less than 10 years (16.3%), 10 to less than 15 years (4.8%), with mean years of experience were 4.12 years. Regarding the mortality rate of assigned patients during their shifts, 18.7% of participants reported experiencing fewer than 50 patient deaths, 55.2% reported between 50 and 100 patient deaths, and 26.1% reported more than 100 patient deaths.

**Table 1:** Distribution of the studied sample according to demographic characteristics (*N* = 417)

Demographic characteristics	*N*	%
**Gender**		
Female	305	73.1
Male	112	26.9
**Age**		
20-<30	359	86.1
30-<40	41	9.8
40-<50	17	4.1
**Mean ± SD**	**26.94 ± 4.96**	
**Marital Status**		
Single	299	71.7
Married	105	25.2
Divorced	4	1.0
Widow	9	2.2
**Residence**		
Urban	250	60.0
Rural	167	40.0
**Living arrangements**		
With family	371	89.0
Alone	20	4.8
With relative	5	1.2
With others	21	5.0
**Numbers of CPR**		
< 50	78	18.7
50–100	230	55.2
> 100	109	26.1
**Years of experiences**		
< 5	302	72.4
5-<10	68	16.3
10-<15	20	4.8
15-<20	7	1.7
20-<25	14	3.4
≥ 25	6	1.4
**Mean ± SD**	4.12 ± 5.24	
**Mortality rate of assigned patients** ^**a**^		
< 50	78	18.7
50–100	230	55.2
> 100	109	26.1

^a^ data exclusively on patients who expired in the ED

Table 2 presents the mean CD-RISC score of participants at 21.12 (SD = 5.43), indicating a moderate ability to cope with stress and adversity. In contrast, the ASDA revealed a mean score of 58.37 (SD = 17.31), suggesting a significant level of anxiety related to death.

**Table 2:** Mean and mean percent of Arabic scale of death anxiety (ASDA) and Connor-Davidson resilience scale (*N* = 417)

Connor-Davidson Resilience Scale (CD-RISC)**	Mean SD
A total score of the CD-RISC	**21.12±5.43**
Arabic scale of death anxiety (ASDA)*	
A total score of the **ASDA**	**58.37±17.31**

The total score of death anxiety on the scale ranged from 20 to 100

*A higher mean score means higher death anxiety

The total resilience score on the scale ranged from 0 to 100

**A higher mean score indicates greater resilience

**Fig. 2:**
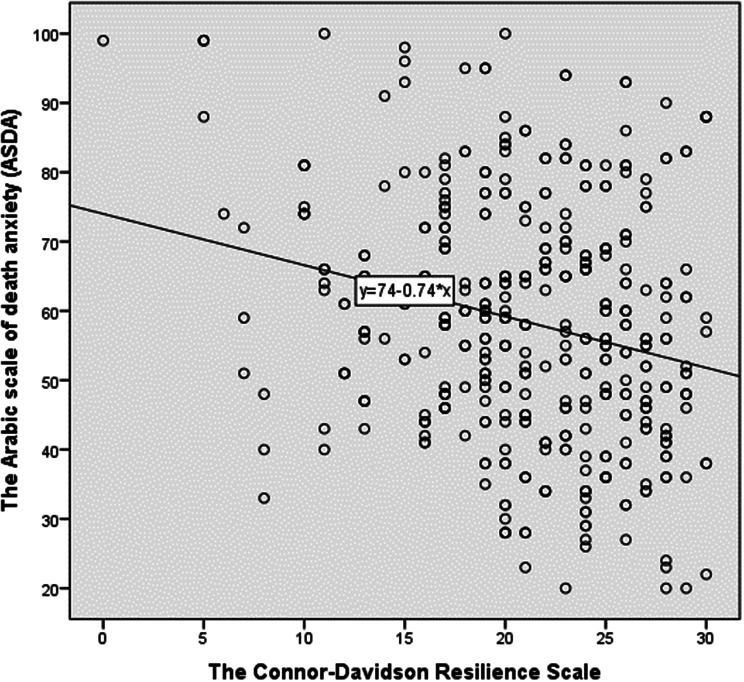
P plots of correlation between the studied variables (*n* = 417)

Figure 2 illustrates the correlation between Connor-Davidson resilience and death anxiety among emergency nurses. A statistically significant negative correlation was found between the two scales (*r* = -0.232, *p* < 0.001), indicating that as levels of resilience tend to increase, levels of death anxiety decrease and vice versa.

Table 3 shows the relationship between demographic characteristics and the ASDA. Females had significantly higher death anxiety scores (59.87 ± 16.99) compared to males (54.28 ± 17.58; *t* = 2.951, *p* = 0.003), indicating greater death anxiety among females. Participants aged 20-<30 years had the highest mean score (59.90 ± 17.00), with significant differences observed between age groups (*F* = 5.696, *p* < 0.001). Urban participants reported higher death anxiety scores (60.15 ± 15.90) than rural participants (55.70 ± 18.96; *t* = 2.500, *p* = 0.013). Significant variations in death anxiety were also noted based on years of experience (*F* = 4.972, *p* < 0.001), with the lowest scores reported among those with 15-<20 years of experience (37.71 ± 20.94). However, no significant differences were found in death anxiety based on marital status, living arrangements, mortality rate, or the number of CPRs performed.

**Table 3:** Relationship between the study variables and socio-demographic data (*N* = 417)

Demographic characteristics	Arabic scale of death anxiety (ASDA)
**Gender**	
Female	59.87 ± 16.99
Male	54.28 ± 17.58
**t (p)**	2.951* (0.003*)
**Age**	
20-<30	59.90 ± 17.00
30-<40	49.02 ± 18.22
40-<50	52.13 ± 10.68
**F (p)**	5.696* (< 0.001*)
**Marital Status**	
Single	59.16 ± 17.23
Married	57.47 ± 17.38
Divorced	40.75 ± 17.04
Widow	50.33 ± 14.83
**F (p)**	2.354 (0.072)
**Residence**	
Urban	60.15 ± 15.90
Rural	55.70 ± 18.96
**t (p)**	2.500* (0.013*)
**Living arrangements**	
With family	58.57 ± 17.23
Alone	55.50 ± 19.25
With relative	49.20 ± 13.14
With others	59.71 ± 17.91
**F (p)**	0.708 (0.548)
**Numbers of CPR**	
< 50	58.97 ± 17.58
50–100	59.68 ± 16.65
> 100	55.17 ± 18.21
**F (p)**	2.593 (0.076)
**Years of experiences**	
< 5	59.78 ± 17.18
5-<10	59.34 ± 15.35
10-<15	52.35 ± 19.34
15-<20	37.71 ± 20.94
20-<25	45.21 ± 14.33
≥ 25	51.17 ± 10.25
**F (p)**	4.972* (< 0.001*)
**Mortality rate of assigned patients** ^**a**^	
< 50	58.97 ± 17.58
50–100	59.68 ± 16.65
> 100	55.17 ± 18.21
**F (p)**	2.593 (0.076)

F: One way ANOVA test t: Student t test *: Statistically significant at *p* ≤ 0.05

^a^ data exclusively on patients who expired in the ED

Table 4 presents a linear regression analysis of significant factors affecting ASDA. Gender, residence, and resilience (as assessed by the Connor-Davidson Resilience Scale) were found to be significant predictors of death anxiety, while age, marital status, years of experience, and mortality rate were not. Specifically, being male was associated with a decrease in death anxiety, with a significant B = -5.335 and Beta = -0.137, meaning males reported lower death anxiety compared to females (*p* = 0.004). Additionally, residence (e.g., rural vs. urban) showed a significant effect, with individuals from specific residences reporting lower death anxiety (B = -3.438, Beta = -0.097, *p* = 0.043). More importantly, higher resilience was strongly associated with lower death anxiety (B = -0.663, Beta = -0.208, *p* < 0.001), indicating that individuals with greater psychological resilience tend to experience less fear of death. In contrast, age (B = -2.457, Beta = -0.071, *p* = 0.403), marital status (B = 1.025, Beta = 0.036, *p* = 0.565), years of experience (B = -2.311, Beta = -0.143, *p* = 0.113), and mortality rate (B = 0.172, Beta = 0.007, *p* = 0.906) did not significantly affect death anxiety. The model explained about 11.7% of the variance in death anxiety (R² = 0.117), with an adjusted R² of 0.102, indicating that while these factors have some influence, other unmeasured variables may be contributing more significantly to death anxiety. The overall model was statistically significant (F = 7.778, *p* < 0.001), highlighting the importance of gender, resilience, and residence in predicting death anxiety among nurses.

**Table 4:** Linear regression analysis showing factors affect Arabic scale of death anxiety (ASDA) (*N* = 417)

Variable	B	Beta	t	*p*	95% CI	
					LL	UL
Age	-2.457	-0.071	-0.837	0.403	-8.223	3.310
Gender (males)	-5.335	-0.137	-2.925*	0.004*	-8.920	-1.750
Residence	-3.438	-0.097	-2.030*	0.043*	-6.767	-0.108
Marital Status	1.025	0.036	0.575	0.565	-2.477	4.527
Years of experiences	-2.311	-0.143	-1.586	0.113	-5.176	0.553
Mortality rate of assigned patients	0.172	0.007	0.118	0.906	-2.695	3.039
Connor-Davidson Resilience Scale	-0.663	-0.208	-4.401*	< 0.001*	-0.959	-0.367
**R**^**2**^ **= 0.117**,** Adjusted R**^**2**^ **= 0.102**, F = 7.778^*^, *p* < 0.001^*^						

F, p: f and p values for the model; R^2^: Coefficient of determination; B: Unstandardized Coefficients

Beta: Standardized Coefficients; t: t-test of significance; LL: Lower limit UL: Upper Limit

*: Statistically significant at *p* ≤ 0.05

## Discussion

This study aimed to examine the relationship between resilience and death anxiety among nurses working in emergency care units. The findings revealed a significant negative correlation between resilience and death anxiety, indicating that higher resilience levels are associated with lower death anxiety. This aligns with the study’s primary objective and underscores the protective role of resilience in mitigating the psychological burden of frequent exposure to death and mortality among emergency nurses.

### Resilience as a protective shield

The moderate levels of resilience and death anxiety observed in this study are consistent with prior research. Resilience, the ability to adapt and thrive in adversity, has been widely recognized as a critical factor in reducing psychological distress among healthcare workers. Mohammadi et al. (2022) and Labrague et al. (2021) similarly found that resilience acts as a buffer against death anxiety and other forms of psychological distress in high-stress healthcare environments [25–27].

Resilience enables individuals to process traumatic events, maintain emotional stability, and uphold professional standards despite repeated exposure to distressing situations. This is particularly critical for emergency nurses, who often face end-of-life scenarios and patient deaths. The ability to process these experiences constructively, as highlighted by Jiang et al. (2022) and Ergin et al. (2021), is essential for sustaining psychological well-being and job performance [28, 29].

The findings also align with the Broaden-and-Build Theory of positive emotions, which posits that resilience and positive emotional experiences broaden individuals’ thought-action repertoires, enabling them to build enduring psychological resources [30]. In emergency nursing, resilience may foster a positive outlook, allowing nurses to view challenges as opportunities for growth rather than insurmountable obstacles. This perspective is supported by Kartal et al. (2022), who found that higher resilience levels were linked to lower thanatophobia (fear of death) among emergency healthcare workers [4].

### Gender differences in death anxiety

The regression analysis identified gender as a significant predictor of death anxiety, with female nurses exhibiting higher levels of death anxiety compared to their male counterparts. This finding is consistent with studies by Jiang et al. (2022) and Xie et al. (2021), which observed similar gender disparities among healthcare workers [28, 31]. The higher levels of death anxiety among female nurses may be attributed to cultural norms that place more significant emotional labor and caregiving responsibilities on women. Additionally, gender-specific coping styles, such as a tendency to internalize stress, may heighten vulnerability to psychological distress [28, 32, 31].

### The role of professional experience in fostering resilience

Younger nurses and those with fewer years of experience reported higher levels of death anxiety, emphasizing the role of professional maturity in fostering emotional resilience. This finding aligns with studies by Magdi (2022) and Ergin et al. (2021), which highlighted that repeated exposure to death-related situations helps nurses develop adaptive coping mechanisms over time [29, 33]. Mentorship programs and structured on-the-job training could be instrumental in supporting early-career nurses as they navigate these challenges, ultimately reducing their death anxiety and enhancing their professional competence. The Stress Exposure Theory further supports this perspective. According to the theory, controlled exposure to stressors, combined with adequate support, helps individuals build resilience by improving their ability to handle future stress. For nurses, experiencing and processing death-related events in a supportive environment may gradually enhance their ability to manage stress in more challenging circumstances. In this sense, professional experience is a key factor in developing emotional resilience, enabling nurses to adapt to the emotional demands of their profession and improve their overall well-being and effectiveness in the workplace [34].

### Urban vs. rural residency

Urban residency emerged as another significant factor influencing death anxiety, with urban nurses reporting higher anxiety levels than their rural counterparts. This may be due to weaker familial and community support systems in urban areas, as well as cultural differences in perceptions of death. In rural settings, stronger social cohesion and cultural practices that view death as a natural transition may mitigate anxiety, as noted in studies exploring rural Egyptian Arab culture [35, 36, 37]. Terror Management Theory (TMT) provides a valuable framework for understanding these cultural influences. According to TMT, cultural worldviews and social connections act as psychological buffers, helping individuals manage existential fears associated with death [38]. Strong cultural beliefs and community ties instill a sense of purpose and belonging, reducing death anxiety and promoting resilience [29]. These findings highlight the importance of culturally sensitive interventions integrating local practices and beliefs about death.

The study’s findings are further supported by the Job Demands-Resources (JD-R) Model, which posits that job demands (e.g., exposure to death) can lead to psychological strain, while job resources (e.g., resilience, social support) can buffer against these effects [39]. In emergency nursing, resilience is a critical personal resource that mitigates the psychological impact of high job demands. This model aligns with the study’s results, emphasizing the importance of enhancing resilience and providing adequate support to reduce death anxiety.

### Limitations of the study

The study utilized a convenience sampling method, which may introduce bias into the sample selection process. The participants were recruited from three specific hospitals in Egypt, which might be different from emergency nurses in other regions or countries. This could limit the generalizability of the findings. The data collection relied on self-reported measures, including death anxiety and resilience scales. Participants may have provided responses that they deemed socially desirable or did not accurately reflect their true feelings and experiences, leading to potential response bias. The study employed a cross-sectional design, which captured data at a single point in time. This design does not allow for examining causal relationships between variables or changes over time. Longitudinal studies would provide more robust evidence regarding the associations between death anxiety, resilience, and other factors among emergency nurses. The study examined the relationship between death anxiety, resilience, and selected demographic variables. However, the analysis did not include other potentially relevant factors, such as coping strategies, social support, and workplace environment. These variables could provide additional insights into the factors influencing nurses’ experiences in emergency care units.

## Conclusion

The study provides compelling insights into the psychological dynamics of emergency nurses, revealing a moderate level of death anxiety alongside a predominance of high resilience among participants. Being female and residing in urban areas were associated with higher odds of experiencing death anxiety, highlighting the influence of gender and environmental factors on psychological well-being. Conversely, higher levels of resilience were strongly associated with lower odds of death anxiety, underscoring the protective role of resilience in buffering against psychological distress.

### Nursing implications

The study underscores the prevalence of death anxiety among emergency nurses, necessitating interventions to address this issue. Healthcare institutions should provide resources and training to help nurses manage death anxiety and enhance resilience skills, such as stress management and mindfulness practices. Support initiatives should be tailored to address the needs of vulnerable groups, like younger or less experienced nurses. A supportive work environment promoting open communication, empathy, and mutual support is essential. Debriefing sessions, counseling services, and team-building activities can help nurses cope with emotional challenges. Nursing education should include training on death and dying, communication skills, and coping strategies. Cultural sensitivity and awareness should be promoted among nursing staff to understand diverse cultural beliefs and practices surrounding death. The study calls for further research to explore the relationship between resilience and death anxiety among emergency nurses, with longitudinal studies potentially providing valuable insights into the effectiveness of interventions aimed at enhancing resilience and reducing death anxiety.

## Electronic supplementary material

Below is the link to the electronic supplementary material.

Supplementary Material 1

Supplementary Material 2

## Data Availability

The datasets used and analyzed during this study are available from the corresponding author upon reasonable request.
