# Supplementary material for: A cross-sectional study on resilience and death anxiety among emergency nurses
Source: BMC Nurs. 2025 Apr 15;24:422. doi: 10.1186/s12912-025-02980-7 (PMC11998161; doi:10.1186/s12912-025-02980-7)
Supplement: Supplementary file 2 — Supplementary Material 2 [file 12912_2025_2980_MOESM2_ESM.pdf]

Geographical Distribution

• **Gender**<br>○ Male<br>○ Female	•**الجنس**<br>• ذكر<br>• أنثى
• **Age**<br>○ 20-<30 years<br>○ 30-<40 years<br>○ 40-<50 years	**الفئة العمرية**<br>• 20إلى أقل من 30 سنة<br>• 30إلى أقل من 40 سنة<br>• 40إلى أقل من 50 سنة
• **Marital Status**<br>○ Single<br>○ Married<br>○ Divorced<br>○ Widowed	•**الحالة الاجتماعية**<br>• أعزب / عزباء<br>• متزوج / متزوجة<br>• مطلق / مطلقة<br>• أرمل / أرملة
• **Place of Residence**<br>○ Urban<br>○ Rural	•**مكان الإقامة**<br>• حضري<br>• ريفي
• **Living Arrangements**<br>○ With family<br>○ Living alone<br>○ With relatives<br>○ Other	•**ظروف السكن**<br>• يعيش مع العائلة<br>• يعيش بمفرده<br>• يعيش مع الأقارب<br>• أخرى
• **Number of CPR (Cardiopulmonary Resuscitation) procedures performed**<br>○ Less than 50 times<br>○ 50-100 times<br>○ More than 100 times	•**عدد مرات إجراء الإنعاش القلبي الرئوي(CPR)**<br>• أقل من 50 مرة<br>• 50إلى 100 مرة<br>• أكثر من 100 مرة
• **Years of Experience**<br>○ Less than 5 years<br>○ 5-<10 years<br>○ 10-<15 years<br>○ 15-<20 years<br>○ 20-<25 years<br>○ 25 years or more	•**سنوات الخبرة**<br>• أقل من 5 سنوات<br>• 5إلى أقل من 10 سنوات<br>• 10إلى أقل من 15 سنة<br>• 15إلى أقل من 20 سنة<br>• 20إلى أقل من 25 سنة<br>• 25سنة فأكثر
• **Number of critically ill patients treated**<br>○ Less than 50 cases<br>○ 50-100 cases<br>○ More than 100 cases	•**عدد المرضى في الحالات الحرجة الذين تم التعامل معهم**<br>• أقل من 50 حالة<br>• 50إلى 100 حالة<br>• أكثر من 100 حالة

Connor-Davidson Resilience Scale (CD-RISC-10)

The 10-item scale comprises ten of the original 25 items from the CD-RISC-10 scale—the total points possible range from 0 to 40.

Possible responses range from:

0 – Not true at all.
1 – Rarely true.
2 – Sometimes true.
3 – Often true.
4 – True nearly all the time.

English	Arabic
I am able to adapt when changes occur.	أقدر أتأقلم/أتكيف لما تحصل أى تغييرات (حولى/فى حياتى).
I can deal with whatever comes my way.	أقدر أتعامل مع أى حاجة تقابلنى.
I try to see the humorous side of things when facing problems.	لما أواجه مشكلات بأحاول أشوف الجانب الفكاهي/الساخر من الأمور.
Having to cope with stress can make me stronger.	قدرتى على التكيف مع الضغوط يمكن أن تجعلني أقوى.
I tend to bounce back after illness, injury or other hardships.	أميل إلى انى آخد خطوة للوراء (أراجع حساباتى) بعد تعرضى للمرض أو الإصابة أو غيرها من الصعوبات.
I believe I can achieve my goals, even if there are obstacles.	أنا مؤمن انى أقدر أحقق أهدافى حتى لو كانت هناك عقبات.
Under pressure, I stay focused and think clearly.	تحت الضغط ، بافضل مركز وأفكر بوضوح.
I am not easily discouraged by failure.	لا أشعر بالإحباط بسهولة بسبب الفشل.
I think of myself as a strong person when dealing with life's challenges and difficulties.	عند التعامل مع تحديات الحياة وصعوباتها باشوف نفسى شخص قوي (بافكر في نفسي كشخص قوي).
I am able to handle unpleasant or painful feelings like sadness, fear, and anger.	باقدر أتعامل مع المشاعر غير السارة (المزعجة) أو المؤلمة مثل الحزن والخوف والغضب.

Death anxiety scale

The scale consists of 20 items, each rated on a Likert scale ranging

1 = No, 2 = A little, 3 = A fair amount, 4 = Much, 5 = Very much.

English	Arabic
I fear death whenever I become ill.	أخاف من الموت عندما يصيبني أي مرض.
I fear looking at the dead.	أخاف من النظر إلى الموتى.
I fear visiting graves.	أخاف من زيارة القبور.
The possibility of having a surgical operation terrifies me.	يقلقني احتمال أن أجري عملية جراحية.
I am afraid of suffering a heart attack.	أخشى الإصابة بنوبة قلبية.
I worry that death may deprive me of someone dear to me.	يقلقني فقدان شخص عزيز عليّ.
I am apprehensive of unknown things after death.	أخاف من المجهول بعد الموت.
I am afraid of looking at a corpse.	أشعر بالضيق عند رؤية جثة.
I fear the torture of the grave.	أخاف من عذاب القبر.
I fear getting a serious disease.	أخشى الإصابة بمرض خطير.
Witnessing the burial procedure terrifies me.	أشعر بالتوتر عند التفكير في مراسم الدفن.
I dread walking in graveyards.	أشعر بعدم الراحة عند السير بين القبور.
I am preoccupied with thinking about what will happen after death.	يزعجني التفكير فيما يحدث بعد الموت.
I am afraid of sleeping and not waking up again.	أخاف أن أموت ولا أستيقظ مجددًا.
The pain accompanying death terrifies me.	يزعجني الألم المصاحب للموت.
I get upset by witnessing a funeral.	أشعر بالقلق عند رؤية جنازة.
The sight of a dying person frightens me.	أشعر بالخوف عند رؤية شخص يحتضر.
Talking about death upsets me.	الحديث عن الموت يجعلني مضطربًا.
I am afraid of getting cancer.	أخشى الإصابة بالسرطان.
I fear death.	أخاف من الموت
